# IL-32: An Emerging Player in the Immune Response Network against Tuberculosis?

**DOI:** 10.1371/journal.pmed.0030274

**Published:** 2006-08-15

**Authors:** Manikuntala Kundu, Joyoti Basu

## Abstract

IL-32 has emerged as an important player in innate and adaptive immune responses. Kundu and Basu discuss a new study in *PLoS Medicine* that explored the role of IL-32 in the context of *M. tuberculosis* infection.

Interleukin-32 (IL-32), which was previously called natural killer cell transcript 4, has recently been recognized as a proinflammatory cytokine (see Glossary) [1]. The main sources of IL-32 are natural killer cells, T cells, epithelial cells, and blood monocytes. Four transcripts of IL-32 are known at present. IL-32 has emerged as an important player in innate and adaptive immune responses, and information is emerging on synergism between IL-32 and other well-characterized players in innate immunity.

The innate immune response depends on the recognition of pathogen-associated molecular patterns by families of pathogen recognition receptors. The best characterized among these are the Toll-like receptor (TLR) [2] and the nucleotide-binding oligomerization domain (NOD) [3] families of proteins. Recent studies have shown that IL-32 synergizes with NOD1 and NOD2 ligands to stimulate IL-1ß and IL-6 release in a caspase-1-dependent manner [4]. These findings are of potential clinical importance in settings where NOD2 plays a protective role, such as in Crohn's disease, where NOD2-dependent production of defensins and cytokines contributes to antimicrobial defense in the gut. These findings are also likely to be of significance in tuberculosis. Individuals homozygous for the 3020ins C NOD2 mutation show a defective cytokine response to *Mycobacterium tuberculosis* [5]. It is in this context that the study reported by Netea et al. in *PLoS Medicine*, in which the authors explored the regulation of IL-32 production by primary cells of the immune system, is of potential importance [6].

## Stimulation of IL-32 by Mycobacteria

Netea et al. explored the role of IL-32 in the context of *M. tuberculosis* infection. In their study, freshly obtained human peripheral blood mononuclear cells (PBMCs) were stimulated with different TLR agonists, and gene expression and synthesis of IL-32 was determined. The authors showed that *M. tuberculosis* could elicit IL-32 release from PBMCs as well as from purifed monocyte populations.

IL-32 has emerged as an important player in innate and adaptive immune responses.

This is the first documented instance of a probable role of IL-32 in the immune response elicited by an intracellular pathogen. The authors also found that other heat-killed organisms, such as *Staphylococcus aureus*, *Candida albicans*, or *Aspergillus fumigatus*, do not stimulate IL-32 production, although these organisms are potent inducers of the proinflammatory cytokines IL-6 and TNF-α.

This study showed that the expression of the genes of isoforms α and γ of IL-32 is stimulated by *M. tuberculosis*, whereas the gene for the ß isoform is constitutively expressed (i.e., always expressed, even without a stimulus). The authors provide evidence in favor of a role of IFN-γ in the induction of IL-32 synthesis. They went on to show that *M. tuberculosis* elicits caspase-1-dependent cleavage of the precursor of IL-18, its release, and IL-18-dependent production of IFN-γ, which could be blocked by IL-18 blocking peptide. The authors also claim that TNF-α release from PBMCs challenged with *M. tuberculosis* occurs in an IL-32-independent manner.

## Strengths and Weaknesses of the Study

The crucial role of IFN-γ in antimycobacterial immunity has been shown in patients with defects in the IFN-γ receptor [7]. Netea and colleagues' findings raise important questions regarding (a) how IL-32 levels vary in patients with defects in IFN-γ receptors who are susceptible to tuberculosis and (b) how these variations may impact the course of the infection. One of the deficiencies of their study stems from the fact that the role of IL-32 in controlling *M. tuberculosis*-dependent cytokine networks has not been investigated directly by neutralizing or blocking IL-32. Rather, the authors' inferences have been drawn indirectly through blocking IFN-γ and IL-18 signaling. PR3 has recently been identified as an IL-32 binding protein [8], and therefore researchers can now explore the effect of neutralizing IL-32 using enzymatically inactive PR3 or PR3-derived peptides.

In addition, the present report fails to address how the synthesis of the different isoforms of the IL-32 protein is regulated, which will require future exploration. IL-32 has been reported to induce the pro-inflammatory cytokines TNF-α and IL-1ß from murine peritoneal macrophages, as well as from phorbol ester-differentiated human THP-1 cells [1]. Netea et al. show that IL-32 production is not associated with TNF-α induction. This raises the question whether the differences in TNF-α induction are attributable to intrinsic differences in behavior of monocytic cells in comparison with differentiated macrophages.

The findings reported in this study raise interesting questions and open avenues for further exploration. One question is whether external stimuli such as IFN-γ regulate IL-32 at both the transcriptional as well as the translational level. The likely roles of the 5′- and 3′-untranslated regions (UTRs) of the IL-32 mRNA in regulating IL-32 production need to be deciphered. IL-32 production has been observed to be TLR independent but IFN-γ dependent [1]. MyD88, a classical TLR adapter protein [2] central to TLR signaling, has recently been shown to signal through the IFN-γ receptor [9] through a non-Toll-IL-1R domain. MyD88 stabilizes mRNA through classical AU-rich elements found in the 3′ UTRs of many mRNAs. Does MyD88 have any role in relation to the production of IL-32? This question is particularly important in view of the report showing fatal *M. tuberculosis* infection in mice in the absence of MyD88 [10]. In general, interactions between 5′ and 3′ UTRs resulting in the formation of an RNA loop increase translational efficiency [11], and RNA-binding proteins act at the level of enhancing or repressing these interactions to control translation. The control processes that regulate translation of IL-32 are additional areas of future exploration.

## Clinical Implications

For the clinician, IL-32 emerges as yet another cytokine whose role in the course of tuberculosis and related infections deserves evaluation. The cellular receptor (or interacting partner) for IL-32 needs to be identified in antigen-presenting cells, which are of particular relevance to tuberculosis. IL-32 contributes to the synovitis of rheumatoid arthritis, and the inflammation of rheumatoid arthritis correlates with IL-32 gene expression [12]. While considering intervention at the level of IL-32 for autoimmune disorders, its role in mounting an effective immune response against invading pathogens also needs to be considered. It would be pertinent to bear in mind that neutralization of IL-32 could render patients more susceptible to tuberculosis. The risk of tuberculosis in patients on anti-TNF-α therapy has already been documented [13].

The most important question raised by these studies is why *M. tuberculosis* induces IL-32 whereas other organisms that are known inducers of IFN-γ do not. The identification of likely additional IL-32 regulating pathways triggered uniquely by mycobacteria deserves immediate attention.

**Glossary**

**Cytokines:** Broad group of signaling proteins that are in general produced by immune cells after cell activation and act as autocrine or paracrine regulators of the immune response.

**Natural killer cells:** Large, granular, bone-marrow-derived lymphocytes of the innate immune system that release cytolytic molecules to kill infected cells and tumor cells.

**PAMPS:** Specific conserved structures of pathogens recognized by pattern recognition receptors on macrophages and dendritic cells.

**Toll-like receptor:** Germline-encoded pattern recognition receptors that recognize conserved molecular patterns shared by microorganisms.

**NOD families of proteins:** A family of cytoplasmic proteins that contain a nucleotide-binding site and a leucine-rich repeat and function as cytosolic sensors involved in innate recognition of microorganisms and regulation of inflammatory responses.

**Interleukins:** Secreted regulatory proteins produced by immune cells such as monocytes and lymphocytes in response to stimuli that help the immune system fight infection and diseases such as cancer.

**Caspase-1 dependent:** Refers to the thiol protease caspase-1-mediated proteolytic processing of the precursor form of IL-18 to the mature, active form of the cytokine.

**Defensins:** Cationic, cysteine-rich peptides found in the cytoplasmic granules of neutrophils and macrophages possessing broad antimicrobial activity against bacteria, fungi, and enveloped viruses.

**PR3:** A granule serine protease present in neutrophils and monocytes capable of processing a variety of biological substrates.

**MyD88:** An adapter molecule that binds to the intracellular domains of TLRs and recruits a number of molecules to the TLR complexes to trigger signaling.

## Abbreviations

IL-32: Interleukin-32
NOD: nucleotide-binding oligomerization domain
PBMC: peripheral blood mononuclear cell
TLR: toll-like receptor
UTR: untranslated region
